# A survey of antimicrobial use practices of Tennessee beef producers

**DOI:** 10.1186/s12917-019-1978-6

**Published:** 2019-07-02

**Authors:** John E. Ekakoro, Marc Caldwell, Elizabeth B. Strand, Lew Strickland, Chika C. Okafor

**Affiliations:** 1 Department of Biomedical and Diagnostic Sciences, College of Veterinary Medicine, University of Tennessee, 2407 River Drive, Knoxville, TN 37996 USA; 2 Department of Large Animal Clinical Sciences, College of Veterinary Medicine, University of Tennessee, 2407 River Drive, Knoxville, TN 37996 USA; 3 Department of Animal Science, University of Tennessee, 2506 River Dr, Knoxville, TN 37996 USA

**Keywords:** Antimicrobial resistance, Antimicrobial use, Survey questionnaires

## Abstract

**Background:**

Inappropriate antimicrobial use (AMU) is a key modifiable factor that leads to the development of antimicrobial resistance (AMR). The objectives of this study were to determine the following among Tennessee beef cattle producers: (1) the opinions on factors driving AMU (2) opinions on alternatives to antimicrobials, (3) the knowledge and perceptions regarding AMU and AMR, and (4) the preferred avenues for receiving information on prudent AMU. A survey questionnaire was made available to participants both in print and online from January 26, 2018 through May 11, 2018. The questions targeted the producers’ demographics and their AMU practices; factors driving producer’s choice of antimicrobials; perceptions, opinions and concerns about AMU and AMR in cattle production. Ordinal logistic regression was used to test for associations between the captured demographic information and producers’ degree of concern about AMR.

**Results:**

Overall, 231 beef producers responded to all or some of the survey questions. More than 60% of the participants mentioned that they kept up-to-date written records on antimicrobial purchases and AMU. Regarding extra-label use, 169 (84.1%) of the 201 respondents did not practice extra-label AMU. Profitability of the beef operation was a key factor influencing the decisions of many producers to use antimicrobials for disease management and prevention on their farms. Of the 228 producers who completed the question on the rating of their degree of concern about AMR, 50 (21.9%) reported that they were very concerned about AMR, 133 (58.3%) were moderately concerned, and 36 (15.8%) reported that they were not concerned about AMR. Nine producers (4%) did not rate their degree of concern about AMR because they were not familiar with what antimicrobial resistance meant. The inferential analyses suggested that younger beef producers were significantly less concerned about AMR when compared to the older ones (*P* = 0.019). Regarding avenues for receiving information on prudent AMU, no single medium was most preferred by all the respondents.

**Conclusions:**

There is a need to promote the use of written antimicrobial treatment protocols among beef producers in Tennessee. Continued training for beef producers on infection prevention and control and prudent AMU is needed.

**Electronic supplementary material:**

The online version of this article (10.1186/s12917-019-1978-6) contains supplementary material, which is available to authorized users.

## Background

Antimicrobial drugs have been described as a common pool resource with the potential to be depleted over time due to the emergence of antimicrobial resistance (AMR) [1]. In beef production, antimicrobials are important to maintain or improve animal health and increase productivity [2]. Although the development of AMR is a complex multifactorial process [3, 4], use of potent broad-spectrum antimicrobials is a key factor selecting for its development [5], and as such, there is increasing concern about the irreparable societal effects of AMR [1, 6, 7]. Globally, the use of bacteriophage-based products, vaccines as well as other infection prevention and control approaches are viewed as promising alternatives to antimicrobials [8–11]. Helke and others [12] suggested that an emphasis on public education, including agricultural education, is critical for facilitating prudent AMU in animal production. Furthermore, a farmer-led approach [13], the whole-of-society approach to antimicrobial effectiveness [7] and One Health approaches to optimization of AMU [14] have been suggested as measures for prolonging the therapeutic life of available antimicrobial drugs.

A collective action towards promoting the prudent/judicious use of antimicrobials is being advocated on a global scale [15]. In the United States, the Food and Drug Administration (FDA) has taken steps to implement its policy on the judicious use of medically important antimicrobial drugs in animals through the Veterinary Feed Directive (VFD) [16]. Researchers [17] have suggested that utilizing approaches appealing to farmers’ internal motivators would increase the success of policy interventions, such as the VFD, that aim to improve AMU. Therefore, understanding current AMU practices of producers and factors that inform and influence those practices is critical for the success of interventions to improve AMU in beef production.

In western Canada, tetracyclines, sulfonamides, and florfenicol were the most commonly used antimicrobials in cow-calf herds during the calving season [18]. Similarly, in Ontario, Canada, a 1999–2002 study found that oxytetracycline, penicillin, macrolides, florfenicol, and spectinomycin were the most commonly used antimicrobials by beef producers [19]. A previous study conducted in 60 cow-calf operations in Tennessee (TN) found that chlortetracycline was the most commonly used antimicrobial in the late 1980s for disease prevention [20]. Additionally, a previous 2007–2008 survey evaluating the producers’ attitudes and practices related to AMU in TN cattle found that approximately 34% of the surveyed population reported using bacterial culture to determine the cause of disease, and 21.5% used culture and susceptibility test results to guide their choice of antimicrobials [21]. The beef 2007–2008 study conducted by USDA reported relatively low levels of AMU on U.S. cow-calf operations [22]. Currently, there is little data published on AMU in beef cattle in the United States. In Europe, AMU data for animals is routinely collected through projects such as the Monitoring of Antimicrobial Resistance and Antibiotic Usage in Animals in the Netherlands (MARAN) and the European Surveillance of Veterinary Antimicrobial Consumption [23]. Routine collection of data on the appropriateness of AMU in animals (including cattle) is needed in the U.S. [23]. A 2014 systematic review [24] found no adequate evidence of a causal relationship between AMU in food animals and the emergence and spread of foodborne AMR-*Campylobacter* and expressed the need for a robust data collection system in the United States that would help identify factors contributing to the persistence of AMR.

This present study was built on the preliminary findings of a previous qualitative study [25] with the aim of exploring how much the results of the qualitative study holds true for the larger population of TN beef producers. This present study, therefore, aimed at contributing to the wider knowledge of AMU by providing insights into the current practices, perceptions, and opinions of TN beef producers regarding AMU and AMR. Specifically, the objectives were to determine the following among Tennessee beef cattle producers: (1) the opinions on factors driving AMU among beef producers, (2) opinions on alternatives to antimicrobials, (3) the knowledge and perceptions regarding AMU and AMR, and (4) the preferred avenues for receiving information on prudent AMU.

## Results

### Participant characteristics and self-reported AMU practices

Of the required sample size of 377 respondents, a total of 231 beef producers (61.3%) participated in the survey. Of the 231 participants, 103 (44.6%) completed the print survey while 128 (55.4%) completed the online version. The estimated response rate for the print survey was 6.3% (103/1629 producers) and 4.7% for the online version (128/2712 producers). The estimated overall survey response rate was 5.3%. Out of the 200 respondents to the question regarding their gender, 35 were females, 163 were males, and two respondents preferred not to report their gender. Complete responses were provided for most questions, with the exception of a few cases where the respondents left some questions unanswered. The demographic information of the respondents is presented in Table 1. No producer mentioned that his/her farm specialized in feedlot operations. More than 60% of the participants mentioned that they kept up-to-date written records on antimicrobial purchases and AMU. Additionally, of the 201 participants who completed the question on extra-label use, 169 (84.1%) did not practice extra-label AMU (Table 2).

**Table 1:** Demographics of beef producers on survey to identify antimicrobial use practices, 2018

Variable	Number (%) of respondents
Gender	**200**
Female	35 (17.5)
Male	163 (81.5)
Preferred not to report gender	2 (1.0)
Age group (years)	**200**
< 30	12 (6.0)
30–39	29 (14.5)
40–49	41 (20.5)
50–59	44 (22.0)
60–69	46 (23.0)
> 70	28 (14.0)
Education level	**202**
< College	47 (23.3)
≥ College	155 (76.7)
Number of years in cattle production	**202**
< 5	23 (11.4)
6–10	19 (9.4)
11–15	17 (8.4)
16–20	24 (11.9)
21–25	24 (11.9)
26–30	21 (10.4)
> 30	74 (36.6)
Beef cattle operation type	**230**
Cow-calf production	171 (74.4)
Backgrounding-stocking	9 (3.9)
Seed-stock operation	6 (2.6)
Multiple operation type and others	44 (19.1)
Herd size	**202**
1–49	84 (41.6)
50–99	54 (26.7)
100–149	28 (13.9)
150–199	12 (5.9)
200–299	13 (6.4)
300–399	5 (2.5)
400–499	1 (0.5)
500+	5 (2.5)
Raised on a cattle farm	**202**
Yes	138 (68.3)
No	64 (31.7)

**Table 2:** Practices of Tennessee beef producers related to antimicrobial use, 2018

Practice	Cattle operation type (number of respondents)	Number of respondents (Row percentage)		
		Yes	Not sure	No
Farm kept up-to-date written records of antimicrobial drug purchases (208 respondents)	Backgrounding-stocking (9)	9 (100)	0 (0)	0 (0)
	Cow-calf production (154)	90 (58)	17 (11.0)	47 (31)
	Multiple operation type, others (39)	26 (66.7)	4 (10.3)	9 (23.0)
	Seed stock operation (6)	6 (100)	0 (0)	0 (0)
	Total	131 (63)	21 (10.1)	56 (26.9)
Farm kept written records of medicated feeds purchased in the framework of VFD (201 respondents)	Backgrounding-stocking (9)	9 (100)	0 (0)	0 (0)
	Cow-calf production (148)	69 (46.6)	21 (14.2)	58 (39.2)
	Multiple operation type, others (38)	25 (65.8)	2 (5.3)	11 (28.9)
	Seed stock operation (6)	6 (100)	0 (0)	0 (0)
	Total	109 (54.2)	23 (11.4)	69 (34.3)
Farm kept up-to-date written records of antimicrobial drugs used to treat animals (209 respondents)	Backgrounding-stocking (9)	9 (100)	0 (0)	0 (0)
	Cow-calf production (155)	102 (65.8)	11 (7.1)	42 (27.1)
	Multiple operation type, others (39)	28 (71.8)	3 (7.7)	8 (20.5)
	Seed stock operation (6)	6 (100)	0 (0)	0 (0)
	Total	145 (69.4)	14 (6.7)	50 (23.9)
Cattle in the farm were sometimes treated with antimicrobials at dosages higher than the label provision (204 respondents)	Backgrounding-stocking (8)	0 (0)	0 (0)	8 (100)
	Cow-calf production (151)	9 (6)	9 (6)	133 (88)
	Multiple operation type, others (39)	6 (15.4)	1 (2.6)	32 (82)
	Seed stock operation (6)	0 (0)	0 (0)	6 (100)
	Total	15 (7.4)	10 (4.9)	179 (87.7)
Farm practiced extra-label AMU (201 respondents)	Backgrounding-stocking (8)	0 (0)	0 (0)	8 (100)
	Cow-calf production (149)	12 (8)	12 (8)	125 (84)
	Multiple operation type, others (38)	7 (18.4)	1 (2.6)	30 (79)
	Seed stock operation (6)	0 (0)	0 (0)	6 (100)
	Total	19 (9.4)	13 (6.5)	169 (84.1)
Farm had written protocols for treating sick animals with antimicrobials (199 respondents)	Backgrounding-stocking (8)	2 (26)	0 (0)	6 (75)
	Cow-calf production (147)	22 (15)	6 (4.0)	119 (81)
	Multiple operation type, others (38)	9 (23.7)	4 (10.5)	25 (65.8)
	Seed stock operation (6)	3 (50)	0 (0)	3 (50)
	Total	36 (18.1)	10 (5)	153 (76.9)

### Objective 1: opinions on factors driving antimicrobial use

Profitability of the beef operation (economic gain from a healthy herd) was a key factor influencing the decisions of many producers to use antimicrobials for disease management and prevention on their farms (141 out of 204 participants [69.1%]). Forty-six (22.5%) participants strongly agreed with the statement “profitability of your operation is an important factor influencing your decision to use antibiotics on your cattle.” Ninety-five (46.6%) agreed, 36 (17.7%) neither disagreed nor agreed with this statement, 20 (9.8%) disagreed, and seven producers (3.4%) strongly disagreed. Regarding the statement “aggressive marketing of antibiotics by pharmaceutical companies greatly influences producers’ use of antibiotics”, 17 (8.3%) out of the 205 respondents strongly agreed with this statement. Eighty-four (41%) respondents agreed, 71 (34.6%) neither disagreed nor agreed with this statement, 26 (12.7%) disagreed, and seven (3.4%) strongly disagreed with this statement.

Of the 28 producers with multiple and other operation types who completed the question on the most common (the number one) disease/condition treated, 14 (50%) mentioned respiratory infections/pneumonia, six (21.4%) mentioned infectious bovine keratoconjunctivitis, two (7.1%) mentioned lameness/hoof problems, and one (3.6%) mentioned anaplasmosis as the most common diseases/conditions for which antimicrobials were used. Four producers in this category mentioned non-specific conditions such as “inflammation (one participant [3.6%])”, “infections (three participants [10.7%])”, and one (3.6%) mentioned “surgical prophylaxis for castration.” For the 99 producers with only cow-calf operation type who completed the question on the most treated (the number one treated) disease/condition with antimicrobials, 24 (24.2%) mentioned respiratory infections/pneumonia, 33 (33.3%) mentioned infectious bovine keratoconjunctivitis, 25 (25.3%) mentioned lameness/hoof problems, three (3%) mentioned “scours”, and two (2%) mentioned anaplasmosis as the most common disease/condition for which antimicrobials were used. Eight (8.1%) producers mentioned other diseases/conditions. Four (4%) producers in this category mentioned they did not have diseases/conditions that required antimicrobials in their farm. For the 7 producers with backgrounding-stocking operation type who completed the question on the most common diseases/conditions treated, five (71.4%) mentioned respiratory infections/pneumonia, two (28.6%) mentioned infectious bovine keratoconjunctivitis. For the five seed stock operators, respiratory infections/pneumonia, infectious bovine keratoconjunctivitis, lameness/hoof problems, anaplasmosis and “warts” were each mentioned by one producer (20%).

Of the 88 cow-calf producers who completed the question on the most used antimicrobial drug for disease management on the farm, 58 (65.9%) participants mentioned drugs belonging to the tetracyclines class, five (5.7%) mentioned penicillins, one (1.1%) mentioned a drug belonging to cephalosporins, 10 (11.4%) mentioned amphenicols, one (1.1%) mentioned a fluoroquinolone, four (4.6%) mentioned macrolides, and one (1.1%) mentioned a sulfonamide. Six respondents (6.8%) had not used antimicrobials on the farm and two (2.3%) were not sure of the antimicrobial most used on the farm. Of the 29 producers with multiple and other operation types, who completed the question on the most used antimicrobial drug for disease management on the farm, 16 (55.2%) participants mentioned drugs belonging to the tetracyclines class, four (13.8%) mentioned penicillins, three (10.3%) mentioned cephalosporins, one (3.5%) mentioned an amphenicol, and four (13.8%) mentioned macrolides. One respondent (3.5%) had not used antimicrobials on the farm. For the seven with backgrounding-stocking, two (28.6%) participants mentioned drugs belonging to the tetracyclines class, one (14.3%) mentioned an amphenicol, three (42.7%) mentioned macrolides, and one (14.3%) had not used antimicrobials on the farm. Of the five seed stock operators who completed this question, four (80%) mentioned tetracyclines and one (20%) mentioned a drug belonging to the macrolide antimicrobial class.

Out of the 226 participants who answered the question on the use of bacterial culture, 128 (56.6%) reported they never used bacterial culture to determine disease cause on their farms; 75 (33.2%) participants reported they sometimes used bacterial culture to determine causes of disease on their farms. Seven (3.1%) respondents reportedly used bacterial culture for disease detection half the time, nine (4%) used bacterial culture for disease detection most of the time, and seven (3.1%) always used bacterial culture for disease detection. Regarding the use of bacterial C/S testing in selecting antimicrobials, 133 (59.4%) participants reported they never used C/S, 61 participants (27.2%) reported that they sometimes used C/S to select antimicrobials, seven (3.1%) about half the time, 13 (5.8%) most of the time, 10 (4.5%) always used C/S.

Regarding who makes the laboratory requests for bacterial culture testing for the farm, 67 of the 91 producers (73.6%) mentioned the veterinarian, 20 (22%) mentioned the producer, and four (4.4%) mentioned the manager. Of the 199 producers who completed the question on whether a veterinarian’s advice was sought before administering antimicrobials, 46 participants (23.1%) mentioned that a veterinarian’s advice was always sought. Fifty-two (26.1%) mentioned that a veterinarian’s advice was sought most of the time, 16 (8%) sought a veterinarian’s advice about half the time, 78 (39.2%) sometimes sought a veterinarians advice on AMU, and seven (3.5%) never sought a veterinarians advice before administering antimicrobials.

### Objective 2: opinions on alternatives to antimicrobials

Additional training of beef producers on infection prevention and control was supported by many survey respondents (152/206 participants [73.8%]). Thirty-three participants (16%) strongly agreed that infection prevention and control measures (farm-level biosecurity and vaccination) would reduce AMU in beef operations. One hundred and nineteen (57.8%) respondents agreed, 38 (18.5%) neither disagreed nor agreed, 15 (7.3%) disagreed, and one (0.5%) strongly disagreed.

### Objective 3: knowledge of and perceptions regarding AMU & AMR

Of the 231 producers, 58 (25.1%) believed there was over-use of antimicrobials in beef production, 92 (39.8%) believed there was no over-use, and 81 (35.1%) were not sure. Regarding the beef production system(s) where antimicrobials were most used, 97 (42%) believed antimicrobials were most used in feedlot operations, 63 (27.3%) in back-grounding stocking, 17 (7.4%) in cow-calf production, five (2.2%) in backgrounding-stocking and feedlot operations, one (0.4%) in seed-stock operation, and 48 (20.8%) were not sure.

The extent to which survey participants were familiar with AMR varied among the 226 respondents to this question. Twenty-five producers (11.1%) reported being extremely familiar with AMR, 59 (26.1%) were very familiar, 97 (42.9%) moderately familiar, 37 (16.4%) slightly familiar, eight (3.5%) not familiar at all. In rating their degree of concern about AMR, of the 228 producers who completed the question on degree of concern about AMR, 50 (21.9%) reported that they were very concerned about AMR, 133 (58.3%) moderately concerned, and 36 (15.8%) reported they were not concerned about AMR. Nine producers (4%) did not rate their degree of concern about AMR because they were not familiar with what antimicrobial resistance meant.

Of the 206 respondents to the statement “some antibiotics you use on your cattle have become ineffective (there is resistance to antibiotics used in cattle)”, twelve producers (5.8%) strongly agreed with this statement. Fifty-four (26.2%) respondents agreed, 84 (40.8%) neither disagreed nor agreed, 48 (23.3%) disagreed, and eight producers (3.9%) strongly disagreed with this statement. Of the 205 respondents to the statement “antibiotic drugs work less effectively than in the past,” eight (3.9%) participants strongly agreed, 43 (21%) agreed, 105 (51.2%) neither disagreed nor agreed, 39 (19%) disagreed, and 10 (4.9%) strongly disagreed.

Additional training of beef producers on prudent AMU was supported by the majority of survey respondents (151 out of 205 participants [73.7%]). Twenty-two producers (10.7%) strongly agreed that producers required additional training on prudent AMU. One hundred and twenty-nine (62.9%) respondents agreed, 37 (18.1%) neither disagreed nor agreed, 15 (7.3%) disagreed, and two (1%) strongly disagreed.

Of the 200 participants who completed the question on antimicrobial drug labels, 149 respondents (74.5%) found antimicrobial drug label instructions easy to understand and interpret while 51 (25.50%) believed antimicrobial drug label instructions were difficult to understand and interpret. All of the 201 survey participants (100%) who responded to the question on the preferred language for antimicrobial label instructions preferred antimicrobial drug labels to be in English. Education level was not significantly associated with producers’ perceptions of difficulty to comprehend antimicrobial label instructions (College/professional vs high school/vocational OR = 1.2; 95% CI = 0.57–2.5; *P* = 0.641).

### Simple associations between demographic variables and producers’ degree of concern about AMR

Producer’s gender (male vs female; *P* = 0.726), being raised on a cattle farm (*P* = 0.461), herd size (*P* = 0.393), education level (*P* = 0.218), number of years in cattle farming (*P* = 0.188), and operation type (*P* = 0.581) were not significantly associated with producer’s degree of concern about AMR. Age was significantly associated (*P* = 0.019) with the producer’s degree of concern about AMR (Table 3) implying that younger producers were significantly less concerned about AMR when compared to the older ones. The age of the beef producer and number of years in cattle farming (r = 0.42, *P* = < 0.0001) were significantly correlated. Based on these simple associations, meaningful multivariable analyses were deemed to be untenable and hence not performed.

**Table 3:** Univariable analyses for associations between various demographic predictors and Tennessee beef producers’ degree of concern about antimicrobial resistant infections, 2018

Variable	Category	OR (95% CI)	*P* Value
Gender	Male vs Female	1.2 (0.5–2.4)	0.726
Raised on a cattle farm	Yes vs No	1.3 (0.7–2.3)	0.461
Herd size	^†^Overall	─	0.393
	50–99 vs 0–49	1.6 (0.8–3.2)	0.193
	50–99 vs ≥ 100	1.5 (0.7–3.3)	0.285
	≥ 100 vs 0–49	1.1 (0.5–2.1)	0.890
Education level	< College vs ≥ College	1.5 (0.8–3)	0.218
Age	^†^Overall	─	0.019
	30–39 vs 40–49	3.3 (1.2–8.9)	0.021
	30–39 vs 50–59	4.4 (1.7–11.9)	0.003
	30–39 vs 60–69	4.3 (1.6–11.5)	0.004
	30–39 vs > 70	6.3 (2–19.8)	0.009
	30–39 vs < 30	1.9 (0.5–7.6)	0.375
	40–49 vs 50–59	1.4 (0.6–3.3)	0.490
	40–49 vs 60–69	1.3 (0.6–3.2)	0.538
	40–49 vs > 70	1.9 (0.7–5.5)	0.215
	< 30 vs 40–49	1.7 (0.4–6.7)	0.426
	60–69 vs 50–59	1 (0.4–2.4)	0.936
	50–59 vs > 70	1.4 (0.5–3.9)	0.499
	< 30 vs 50–59	2.4 (0.6–9)	0.205
	60–69 vs > 70	1.5 (0.5–4)	0.456
	< 30 vs 60–69	2.3 (0.6–8.7)	0.223
	< 30 vs > 70	3.4 (0.8–14.3)	0.101
Number of years in cattle farming	^†^Overall	─	0.188
	6–10 vs < 5	2.3 (0.6–8.1)	0.208
	6–10 vs 11–15	3.8 (1–14.3)	0.052
	6–10 vs 16–20	1.4 (0.4–5)	0.574
	6–10 vs 21–25	0.8 (0.2–2.9)	0.761
	6–10 vs 26–30	1.7 (0.5–6.2)	0.429
	6–10 vs > 30	2.4 (0.8–7)	0.107
	11–15 vs < 5	0.6 (0.2–2.1)	0.428
	11–15 vs 16–20	0.4 (0.1–1.3)	0.132
	11–15 vs 21–25	0.2 (0.1–0.8)	0.018
	11–15 vs 26–30	0.5 (0.1–1.6)	0.225
	11–15 vs > 30	0.6 (0.2–1.8)	0.408
	16–20 vs < 5	1.6 (0.5–5.2)	0.451
	16–20 vs 21–25	0.6 (0.2–1.9)	0.354
	16–20 vs 26–30	1.2 (0.3–4)	0.792
	16–20 vs > 30	1.7 (0.6–4.4)	0.292
	21–25 vs < 5	2.8 (0.8–9)	0.096
	21–25 vs 26–30	2.1 (0.6–6.9)	0.248
	21–25 vs > 30	2.9 (1.1–7.8)	0.031
	26–30 vs < 5	1.3 (0.4–4.6)	0.641
	26–30 vs > 30	1.4 (0.5–4)	0.493
	> 30 vs < 5	0.9 (0.4–2.5)	0.897
Cattle operation type	Cow-calf vs Multiple operation and others	1.2 (0.6–2.5)	0.581

^†^Overall = overall effect of predictor on outcome variable

### Objective 4: avenues for receiving information on prudent AMU

Regarding avenues for receiving information on prudent AMU, no single medium was most preferred by all the respondents. Of the 196 producers who responded to the question on avenues for receiving information on prudent AMU, 19 (9.7%) participants preferred brochures, 71 (36%) participants preferred educational seminars, five (2.6%) participants preferred videos, five (2.6%) mentioned flow charts for the ban, three (1.5%) participants mentioned laminated posters, 37 (19%) participants mentioned a producers’ handbook on prudent AMU, and 39 (19.9%) mentioned combinations of avenues such as videos on prudent AMU, brochures and educational seminars. Seventeen (8.7%) participants chose the “others” option and mentioned avenues such as the veterinarian, drug label instructions. Of the 202 participants who answered the question on the preferred language for receiving information on prudent AMU, 200 (99%) preferred to receive AMU information in English.

## Discussion

The findings of this survey are generally in keeping with the findings of our previous qualitative study of AMU among TN beef producers [25] and provide insight into the AMU practices of TN beef producers. Additionally, this present study identified opportunities for improving AMU among these producers at a time when AMU in food animals is under public scrutiny. Results of this study suggest that extra-label AMU among TN beef producers could be very low. Written AMU protocols could reduce treatment errors since most of antimicrobial treatments in farms are often administered by non-technical farm personnel (the farmer or farm employees) [26, 27]. In the present study, the majority of the respondents (76.9%) mentioned that their farms did not utilize written protocols for treating sick animals with antimicrobials, suggesting a need for veterinarians and TN beef Extension agents to emphasize and encourage the development and use of written AMU protocols. Similar to the findings of two Canadian studies [18, 19] where tetracyclines and florfenicol were the most commonly used antimicrobials, 65.9% of the cow-calf producers in the present study mentioned tetracyclines (e.g. oxytetracycline) and 11.4% mentioned amphenicols (e.g. florfenicol) as the most used antimicrobial drug for disease management on the farm.

Although a large proportion (37.2%) of producers in the present study were either extremely familiar or very familiar with AMR, many (19.9%) were either slightly familiar or not familiar at all with AMR, suggesting a need for more education on AMR and AMU. Moreover from the univariable analyses, producers in the 30–39 age group were significantly less concerned about AMR when compared to those in the 40–49, 50–59, 60–69, and > 70 age groups. Possibly this results may reflect a lack of awareness of the consequences of AMR among producers in the 30–39 years age group. It is also possible that producers in the 30–39 years age-group rarely participate in educational programs related to AMR when compared to those in other age groups and, as such, could be less informed about AMR and its consequences. An awareness campaign on AMR targeting producers in the 30–39 years age-group could be beneficial. It is important to note that a meaningful multivariable analyses was deemed untenable, perhaps because some important predictors of the producer’s degree of concern about AMR were not measured/included in the study.

In the present study, 63% of the surveyed producers kept written records of antimicrobial drug purchases and 69.4% kept written records of antimicrobial drugs used to treat animals, whereas in the 2007/2008 survey of TN beef producers, 39.4% of the surveyed producers kept records of antimicrobial purchases and 32.2% kept records of AMU [21]. The findings of the present study suggest there was an increase in the number of TN beef producers keeping records on antimicrobial purchases and AMU over the last 10 years. This increase in record keeping could reflect an increased awareness of the importance of farm record keeping among beef producers. Similarly, compared to the 2007/2008 survey findings where 13.5% of producers treated their cattle with antimicrobials at dosages higher than the label instructed, the findings of this present study showed that only 7.3% of the surveyed producers mentioned that they sometimes treated their cattle with antimicrobials at dosages higher than the label provision. This finding suggests that producers’ practice of treating animals with antimicrobials at higher dosages contrary to the label indication may have dropped by half (50%) over the past 10 years. This drop could be due to the producers’ recognition of the importance of adhering to label instructions or due to the improvement in producers’ knowledge of AMU.

In the present study, 56.6% of the participants reported they never used bacterial culture to determine disease cause on their farms and 59.4% of the participants mentioned they never used C/S in selecting antimicrobials. These findings generally suggest that, although reportedly practiced in some beef farms, the use of bacterial culture to determine disease cause and the use of C/S tests for antimicrobial selection is currently not widely practiced on TN beef farms. A 2007–2008 survey [21] found that 34% of producers used bacterial culture to determine disease cause and 31.5% of the surveyed beef producers reported using C/S to choose antimicrobials. Compared to the 2007/2008 survey, the findings reported in the present study suggest that there has not been any significant change (increase) in the use of C/S test results among TN beef producers over the last 10 years. Possibly, many producers have not adopted the use of C/S due to cost implications or lack of awareness about the benefits of C/S. At herd level, routine C/S can be useful for detecting changes in pathogen susceptibility and herd antimicrobial response, and for re-evaluating antimicrobial treatment options [22]. Again, veterinarians and TN beef Extension agents should create more awareness regarding the benefits of C/S among TN beef producers. Such awareness especially from the herd veterinarian (where applicable).

A previous review [28] identified farmers’ belief that AMU will improve profitability as a barrier to sustainable AMU because it hinders the reduction of AMU. In east Asia, farm profitability, disease prevention and mortality rate reduction were identified as drivers of AMU in livestock [29]. In the present study, 69% of the producers agreed that profitability of the beef operation (economic gain) was a key factor influencing the decisions of many producers to use antimicrobials in their farms. This finding is not surprising given that the risk of disease transmission may exert significant economic pressure on producers to use antimicrobials for infectious disease management and prevention [2]. However, producers need to be informed that profitability can be realized with minimal or no AMU, if appropriate infection prevention and control measures are implemented on the farm. The 1986 antimicrobial growth promoters (AMGP) ban in Sweden showed that it is possible for farmers to achieve good and competitive production under good on-farm production systems [30]. This ban on AMGP and more focus on disease prevention and correct AMU significantly reduced total AMU in Sweden.

It is a common practice in many countries for pharmaceutical company representatives to directly market antimicrobials to farmers. The marketing of antimicrobials directly to food animal producers is discouraged by the World Organization for Animal health [31]. Similar to the findings of our previous qualitative study, where producers believed that antimicrobial marketing techniques are persuasive and aggressive [25], our findings in the present study show that many producers (41%) believed the aggressive marketing of antibiotics by pharmaceutical companies greatly influenced producers’ AMU. Many producers (25.5%) in the present study believed that antimicrobial drug label instructions were difficult to understand and interpret. Although this finding may not be generalized to the entire United States beef producer population, it suggests that veterinary pharmaceutical companies should consider labeling antimicrobial drugs in non-technical, easy-to-understand language for increased comprehension among producers. A countrywide investigation of the perceptions among beef producers about current antimicrobial labels and information on the antimicrobial package inserts may prove useful.

In the present study, no single medium/avenue for receiving AMU information was most preferred by all producers. This finding confirms the findings of previous studies, where farmers differed in their preference for receiving information on management and infection/disease prevention and control [17]. Previous scholars have suggested that veterinarians should act as the main information source for farmers on AMU because they are perceived as trustworthy social referents for farmers [32]. The U.S. Food and Drug Administration (FDA) guidelines [33] states that “use of medically important antimicrobial drugs in food-producing animals should be limited to those uses that include veterinary oversight or consultation.” In the present study, 46 participants (23.1%) mentioned that a veterinarian’s advice was always sought before antimicrobials were administered and 52 (26.1%) mentioned a veterinarian’s advice was sought most of the time. It could be beneficial for policy interventions towards prudent AMU to channel AMU-related behavioral change messages to beef producers through veterinarians, where possible. Furthermore, targeted behavioral change messages towards prudent AMU should be integrated into routine veterinary farm visits and beef Extension training programs. Behavioral techniques, such as motivational interviewing informed by assessing producers’ readiness for change, could be used [34]. Additionally, these behavioral change messages could be packaged for beef producers in the form of brochures, a producer’s handbook on prudent AMU or prudent AMU videos. Educational seminars should be used to identify AMU training needs and raise more awareness about AMR and prudent AMU among beef producers. However, scholars in Europe suggested that providing a sense of ownership of the recommendations for judicious AMU [32] and farmer-led approaches [13] can be useful in causing behavioral change among producers. Exploring appropriate methods for quantifying on-farm AMU in the U.S., may be invaluable since such measures could cause behavioral change towards prudent AMU.

The strength of the present study was that preliminary findings from our previous qualitative study were used in developing the survey questionnaire. Nevertheless, it is possible that the results of this study could have been influenced by social desirability bias, which is a form of response bias in which respondents provide socially desirable answers to survey questions [35]. Socially desirability bias, if any, could be minimal because the survey was voluntary, anonymous and self-administered. The respondents were assured of the anonymity of their responses in the informed consent statement and the self-administration of the survey provided the respondents with adequate privacy to provide honest responses. Additionally, as a limitation, selection bias could be an issue. However, selection bias could be minimal because the demographic characteristics of late respondents and their responses to survey questions were similar when compared with early respondents [36], suggesting the survey answers of the respondents could be similar to those of non-respondents. The overall response rate for the present study was 5.3%. This is not surprising because securing a high number of responses to a survey can be practically difficult [37]. The observed overall response rate could be due to the fatigue associated with over-surveying leading to the reluctance of respondents to complete and return the questionnaire [38]. Furthermore, our actual sample size (231) was lower than the expected sample size (377) by almost 40% (despite the concerted efforts to realize our expected sample size). This could have reduced the power of our study. Nevertheless, a post hoc evaluation of the effect of the sample size on the study’s margin of error and confidence level showed that the margin of error in our study increased from 5 to 6.4% and our confidence level decreased from 95 to 87.3%. In summary, 6.4% of the time, we would expect our obtained survey responses to be more than the margin of error away from the true answer and there is only 87.3% chance that our obtained responses are within the margin of error of the true answer.

## Conclusions

The proportion of TN beef producers keeping farm records on antimicrobial purchases and AMU may have increased over the last 10 years. The proportion of beef producers treating cattle with antimicrobials at dosages higher than the label indication may have reduced by 50% over the last 10 years. Culture and sensitivity tests for antimicrobial selection are currently not widely used in TN beef farms, perhaps due to cost implications. There is need to promote the use of written antimicrobial treatment protocols among TN beef producers. Continued training for beef producers on infection prevention and control, and prudent AMU is needed.

## Materials and methods

### Study design and administration of survey

This study targeted beef producers in the U.S. state of TN. With an assumed TN beef producer population size of 20,000 and a 50% response distribution, 377 participants were determined to be the appropriate sample size for this study at 95% confidence level and a margin of error of 5%. A questionnaire consisting of a section for beef producers and a section for dairy producers was developed and evaluated by two professionals with expertise in AMU to ensure all critical issues were identified and covered (see additional file 1 in the supporting information for the survey questionnaire). Participants whose primary cattle production was beef, were required to complete the beef producer section of the questionnaire. The data obtained from five beef focus groups previously conducted by the authors [25] was used to develop the questionnaire. The University of Tennessee Knoxville, Institutional Review Board for the Protection of Human Subjects in Research approved the study (Protocol number: UTK IRB-17- 03884-XP) and a written consent to participate was obtained prior to taking the survey. The 56 survey questions targeted the producers’ demographics and their AMU practices, factors driving producer’s choice of antimicrobials, and perceptions, opinions, and concerns about AMU and AMR in cattle production. Three-point scales and ordinal Likert scales were used to capture participant responses to the survey questions.

The targeted producer demographic information included age, sex (male versus female), level of education, herd size, whether the producer raised on a livestock farm, and number of years in cattle farming. These demographic data were our explanatory variables of interest. Our main outcome of interest was the producers’ degree of concern about antimicrobial resistant (AMR) infections in cattle. The producers’ degree of concern about AMR was captured using a three-point scale (not concerned, moderately concerned, and very concerned). Also, the association between levels of education and producers’ perception of antimicrobial label instructions was of interest.

The survey questionnaire was made available to participants both in print and online. One producer per farm received a single questionnaire and the survey responses from each participant represent attributes of a unique farm. Producers who completed the print questionnaire were requested not to complete the online survey and vice versa in the informed consent statement. Qualtrics software (Provo, UT) housed the on-line version of the survey, which was adapted for computer, tablet, and cell phone responses. Participant responses were de-identified using the anonymize function in Qualtrics such that no personal information was collected. In an effort to achieve our desired sample size, beef producers were notified during the Tennessee Cattle Men’s Association (TCA) annual meeting in January 2018, that the online survey would be sent to them via email. Subsequently, all 2,712 producers on the TCA mailing list received an email invitation to take the survey. Additionally, an anonymous survey link and QR code for the online survey were provided to the TCA vice president for distribution to producers willing to take the survey. Follow-up email reminders were sent to non-respondents of the on-line survey every two weeks.

The printed questionnaire was distributed to producers attending the TCA annual meeting and producer Extension meetings across the state. Completed printed questionnaires were returned to the investigators or mailed to the last author. Both the printed and online survey remained open from January 26, 2018, through May 11, 2018. Participation in the survey was voluntary. All participants were invited to participate in a $10 gift card raffle taken at the end of the survey and the winners were randomly selected. Eligibility to participate in the raffle was not contingent upon survey completion.

### Statistical analysis

Descriptive statistics (frequencies and proportions) were used to summarize the data (see additional file 2 for the raw data). The most treated disease/condition and the most used antimicrobials (mentioned as generic or trades names), that were captured as free text from the producer responses were further grouped into classes as described previously [39]. A commercial statistical software (SAS, version 9.4, SAS Institute Inc., Cary, NC) was used to perform the descriptive and inferential analyses and no corrections were made on missing data. Another commercial software (Tableau software, version 8.2, Seattle, WA) was used to create stacked bar charts for responses on the Likert scales.

Univariable analyses were performed using ordinal logistic regression to test for associations between the captured demographic information and the producers’ degree of concern about AMR. For the univariable analyses, level of education was reclassified into two categories, < college or ≥ college. The category ≥ college included producers with undergraduate and graduate level education. Herd size was reclassified into three categories 0–49, 50–99, and > 100 beef cattle, and age was reclassified into < 30, 30–39, 40–49, 50–59, 60–69, and ≥ 70 using the quantile classification method. The variable number of years in cattle production referred to the number of years a producer had spent in cattle farming and not necessarily the longevity of the farm. In these analyses, the probabilities modeled were cumulated over the lower ordered values (the probability of a beef producer being less concerned about AMR was modeled). A multivariable ordinal logistic regression model was not fitted because it was deemed untenable based on the findings from the univariable analyses.

## Additional files

Additional file 1: Survey questionnaire. (DOCX 29 kb)

Additional file 2: Raw data set. (XLSX 100 kb)

## Data Availability

The raw data pertaining to the manuscript is provided in additional file 2.

## Abbreviations

AMR: Antimicrobial resistance
AMU: Antimicrobial use
C/S: Culture and sensitivity
FDA: Food and Drug Administration
USDA: United States Department of Agriculture
VFD: Veterinary Feed Directive
