# Supplementary material for: A survey of antimicrobial use practices of Tennessee beef producers
Source: BMC Vet Res. 2019 Jul 2;15:222. doi: 10.1186/s12917-019-1978-6 (PMC6604443; doi:10.1186/s12917-019-1978-6)
Supplement: Supplementary file 1 — Survey questionnaire. (DOCX 29 kb) [file 12917_2019_1978_MOESM1_ESM.docx]

Antibiotic use practices of cattle producers,Tennessee

Start of Block: Block 1

Q1 Thank you for participating in this study. Please read the informed consent statement before proceeding by clicking on this link.  (Informed Consent Statement will open in a new window or tab.)
 Approved Informed consent statement

Q2 I have read and understood the information in the above informed consent statement. Please choose the option below that best represents your consent.

- I agree to participate in the survey and have the chance to win $10 Wal-Mart gift card ( If checked, branches to survey and then to separate link to raffle drawing). (1)
- I do not agree to participate in the survey, but I want to participate in the raffle for $10 Wal-Mart gift card (If checked, branches to a separate link to raffle drawing). (2)
- I do not agree to participate in the survey and I do not want to participate in the raffle for $10 Wal-Mart gift card (If checked, branches to thank you page). (3)

End of Block: Block 1

Start of Block: Default Question Block

Q3 Which of the following best describes your primary cattle production?

- Beef production (1)
- Dairy production (2)
- Other (specify) (3) ________________________________________________

Skip To: Q10 If Which of the following best describes your primary cattle production? = Beef production

Q4 How are antibiotic-treated cows distinguished from the rest of the milking herd at milking?

- Milked with a separate milking unit (1)
- All cows are milked using the same milking unit (2)

Q5 When are antibiotic-treated cows milked?

- Milked first (1)
- Milked in-between (2)
- Milked last (3)

| Page Break |  |
| --- | --- |

Q6 Are cows routinely screened after freshening for antibiotics with an antibiotic residue detection test?

- No (1)
- Not sure (2)
- Yes (3)

Q7 Were there any antibiotic residue violations in your milk in the past 6 months?

- Yes (1)
- Not sure (2)
- No (3)

Q8 To what extent do you agree or disagree with the following statement? " If all dairy producers in Tennessee followed best practices **in the use of antibiotics**, overall use of antibiotics in Tennessee dairy cattle would decrease".

- Strongly disagree (1)
- Disagree (2)
- Neither agree nor disagree (3)
- Agree (4)
- Strongly agree (5)

| Page Break |  |
| --- | --- |

Q9 To what extent do you agree or disagree with the following statement? " If all dairy producers in Tennessee followed **best milk quality practices**, overall use of antibiotics in Tennessee dairy cattle would decrease".

- Strongly disagree (1)
- Disagree (2)
- Neither agree nor disagree (3)
- Agree (4)
- Strongly agree (5)

Display This Question:

If Which of the following best describes your primary cattle production? = Beef production

Q10 Which of the following best describes your beef production system? (check all that apply).

- Cow-calf production (1)
- Backgrounding-stocking (2)
- Feedlot operations (3)
- Seed stock (4)
- Other (specify) (5) ________________________________________________

Display This Question:

If Which of the following best describes your primary cattle production? = Beef production

Q11 Do you think there is over-use of antibiotics in beef production?

- Yes (1)
- Not sure (2)
- No (3)

| Page Break |  |
| --- | --- |

Display This Question:

If Which of the following best describes your primary cattle production? = Beef production

Q12 In which of the following beef production systems, are antibiotics used most?

- Cow-calf production (1)
- Backgrounding-stocking (2)
- Feedlot operations (3)
- Not sure (4)
- Other (specify) (5) ________________________________________________

Display This Question:

If Which of the following best describes your primary cattle production? = Beef production

Q13 Were there any antibiotic residue violations in beef (meat products) from cattle raised on your farm last year?

- Yes (1)
- Not sure (2)
- No (3)

Display This Question:

If Which of the following best describes your primary cattle production? = Beef production

Q14 To what extent do you agree or disagree with the following statement? " If all beef producers followed best practices **in the use of antibiotics**, overall use of antibiotics in Tennessee beef cattle would decrease".

- Strongly disagree (1)
- Disagree (2)
- Neither agree nor disagree (3)
- Agree (4)
- Strongly agree (5)

| Page Break |  |
| --- | --- |

Display This Question:

If Which of the following best describes your primary cattle production? = Beef production

Q15 To what extent do you agree or disagree with the following statement? " If all beef producers followed best practices **in the management of their herds**, overall use of antibiotics in Tennessee beef cattle would decrease".

- Strongly disagree (1)
- Disagree (2)
- Neither agree nor disagree (3)
- Agree (4)
- Strongly agree (5)

Q16 How familiar are you with the Veterinary Feed Directive (VFD)?

- Not familiar at all (1)
- Slightly familiar (2)
- Moderately familiar (3)
- Very familiar (4)
- Extremely familiar (5)

Q17 What is your opinion about the Veterinary Feed Directive (VFD)?

- I am not familiar with VFD (1)
- VFD is not useful (2)
- VFD is neither useful nor beneficial (3)
- VFD is somewhat useful (4)
- VFD is very useful (5)

| Page Break |  |
| --- | --- |

Q18 Were you aware of the Veterinary Feed Directive (VFD) before its implementation?

- Yes (1)
- Not sure (2)
- No (3)

Q19 How has the Veterinary Feed Directive (VFD), from the time it became effective, influenced your use of veterinary services?

- VFD has reduced my use of veterinarian services (1)
- VFD has not influenced me to seek veterinarian services (2)
- VFD has caused me to seek veterinarian services more frequently (3)
- Other (specify) (4) ________________________________________________

Q20 Antibiotic resistance is the ability of bacteria (disease causing germs) to resist or be unaffected by the effects of medication. How familiar are you with the subject of antibiotic resistance?

- Not familiar at all (1)
- Slightly familiar (2)
- Moderately familiar (3)
- Very familiar (4)
- Extremely familiar (5)

| Page Break |  |
| --- | --- |

Q21 How do you rate your degree of concern about antibiotic-resistant infections in cattle production?

- I am not familiar about antibiotic-resistant infections in cattle production (1)
- Not concerned (2)
- Moderately concerned (3)
- Very concerned (4)

Q22 How often do you observe antibiotic drug withdrawal times in your farm?

- Never (1)
- Sometimes (2)
- About half the time (3)
- Most of the time (4)
- Always (5)

Q23 How often do you use bacterial culture to determine the cause of disease on your farm?

- Never (1)
- Sometimes (2)
- About half the time (3)
- Most of the time (4)
- Always (5)

| Page Break |  |
| --- | --- |

Q24 How often do you use bacterial culture to select the most appropriate antibiotics to use on your farm?

- Never (1)
- Sometimes (2)
- About half the time (3)
- Most of the time (4)
- Always (5)

Skip To: Q26 If How often do you use bacterial culture to select the most appropriate antibiotics to use on your... = Never

Q25 Who makes the laboratory request for bacterial culture testing for your farm?

- The producer (1)
- The manager (2)
- The veterinarian (3)
- Other (specify) (4) ________________________________________________

| Page Break |  |
| --- | --- |

Q26 Thinking about your practices **BEFORE** the Veterinary Feed Directive-final rule became effective on January 1, 2017, how important were the following factors in determining the choice of antibiotics used in your farm?

|  | Not at all important (1) | Slightly important (2) | Moderately important (3) | Very important (4) | Extremely important (5) |
| --- | --- | --- | --- | --- | --- |
| Recommendations from other producers (1) |  |  |  |  |  |
| Clinical signs and symptoms (2) |  |  |  |  |  |
| Cost of the antibiotic (3) |  |  |  |  |  |
| Veterinarian's prescription (4) |  |  |  |  |  |
| Positive culture and susceptibility tests (5) |  |  |  |  |  |
| Your farming experience (6) |  |  |  |  |  |
| Drug withdrawal times (7) |  |  |  |  |  |
| Recommendations from pharmaceutical company representatives (8) |  |  |  |  |  |
| Recommendations from feed mill operatives (9) |  |  |  |  |  |
| Availability of antibiotic(s) (10) |  |  |  |  |  |
| The ability of the drug to cure the infections (11) |  |  |  |  |  |
| Concerns for animal welfare (12) |  |  |  |  |  |
| Concerns for food security (13) |  |  |  |  |  |

| Page Break |  |
| --- | --- |

Q27 Thinking about your practices **AFTER** the Veterinary Feed Directive-final rule became effective on January 1, 2017, how important are the following factors in determining the choice of antibiotics used in your farm?

|  | Not at all important (1) | Slightly important (2) | Moderately important (3) | Very important (4) | Extremely important (5) |
| --- | --- | --- | --- | --- | --- |
| Recommendations from other producers (1) |  |  |  |  |  |
| Clinical signs and symptoms (2) |  |  |  |  |  |
| Cost of the antibiotic (3) |  |  |  |  |  |
| Veterinarian's prescription (4) |  |  |  |  |  |
| Positive culture and susceptibility tests (5) |  |  |  |  |  |
| Your farming experience (6) |  |  |  |  |  |
| Drug withdrawal times (7) |  |  |  |  |  |
| Recommendations from pharmaceutical company representatives (8) |  |  |  |  |  |
| Recommendations from feed mill operatives (9) |  |  |  |  |  |
| Availability of antibiotic(s) (10) |  |  |  |  |  |
| The ability of the drug to cure the infections (11) |  |  |  |  |  |
| Concerns for animal welfare (12) |  |  |  |  |  |
| Concerns for food security (13) |  |  |  |  |  |

| Page Break |  |
| --- | --- |

Q28 To what extent do you agree or disagree with the following statements?

|  | Strongly disagree (1) | Disagree (2) | Neither disagree nor agree (3) | Agree (4) | Strongly agree (5) |
| --- | --- | --- | --- | --- | --- |
| The Veterinary Feed Directive (VFD) has limited your access to antibiotics (1) |  |  |  |  |  |
| Most veterinarians do not know how to write VFD prescriptions (2) |  |  |  |  |  |
| VFD needs to be updated to accommodate current flaws in execution (3) |  |  |  |  |  |
| My veterinarian can write an accurate VFD prescription (4) |  |  |  |  |  |
| VFD has introduced additional costs of involving a veterinarian (5) |  |  |  |  |  |
| You are aware of how to properly dispose any unused feed from the VFD (6) |  |  |  |  |  |
| The VFD would lead to increased use of injectable antibiotics by producers (7) |  |  |  |  |  |
| The VFD has created more black-market access to in feed antibiotics by producers (8) |  |  |  |  |  |
| The VFD has increased the costs of feed (9) |  |  |  |  |  |
| The VFD has negatively affected small scale producers (10) |  |  |  |  |  |
| The VFD has set cattle producers up for financial loss because it has removed access to preventive in-feed medicines (11) |  |  |  |  |  |
| The VFD is useful for producing safer food (12) |  |  |  |  |  |

| Page Break |  |
| --- | --- |

Q29 To what extent do you agree or disagree with the following statements?

|  | Strongly disagree (1) | Disagree (2) | Neither disagree nor agree (3) | Agree (4) | Strongly agree (5) |
| --- | --- | --- | --- | --- | --- |
| Producers require additional training on prudent use of antibiotics (1) |  |  |  |  |  |
| Aggressive marketing of antibiotics by pharmaceutical companies greatly influences producers' use of antibiotics (2) |  |  |  |  |  |
| Training producers on infection control (bio-security) and vaccination would reduce the use of antibiotics (3) |  |  |  |  |  |
| Some antibiotics you use on your cattle have become ineffective (there is resistance to antibiotics used in cattle) (4) |  |  |  |  |  |
| Profitability of your operation is an important factor influencing your decision to use antibiotics on your cattle (5) |  |  |  |  |  |
| Antibiotic drugs work less effectively than in the past (6) |  |  |  |  |  |

| Page Break |  |
| --- | --- |

Q30 Where do you buy antibiotics drugs for your farm?

- From internet sites (1)
- Over-the- counter (local Cooperative) (2)
- From a veterinarian (3)
- Directly from a distributor (pharmaceutical Company Representative) (4)
- Directly from a drug company (5)
- Other (specify) (6) ________________________________________________

Q31 What criteria are used on the farm to determine the need for antibiotics treatment of sick animals?

- Clinical signs and symptoms (1)
- Positive culture and sensitivity tests (2)
- Other (specify) (3) ________________________________________________

Q32 Does your farm keep up-to-date written records of antibiotic drug purchases?

- No (1)
- Not sure (2)
- Yes (3)

| Page Break |  |
| --- | --- |

Q33 Does your farm keep written records on medicated feeds purchased in the frame work of the veterinary feed directive?

- No (1)
- Not sure (2)
- Yes (3)

Q34 Does your farm keep up-to-date written records of antibiotic drugs used to treat animals?

- No (1)
- Not sure (2)
- Yes (3)

Q35 How often does your veterinarian visit your farm?

- Never (1)
- On routine calls (2)
- As needed (3)

| Page Break |  |
| --- | --- |

Q36 In what format do you receive prescriptions (and other advice) from your veterinarian?

- Through Telephone conversation (1)
- Through text messages (2)
- Through e-mail (3)
- Through face-to-face interactions during farm visits (4)
- Social media (5)
- Other (specify) (6) ________________________________________________

Q37 What are the 5 antibiotic drugs that you commonly use on your farm? (Rank from the most to the least)

- 1. Most used (1) ________________________________________________
- 2 (2) ________________________________________________
- 3 (3) ________________________________________________
- 4 (4) ________________________________________________
- 5. Least used (5) ________________________________________________

Q38 What are the 5 diseases/conditions that you commonly treat with antibiotics on your farm? (Rank from the most to the least)

- 1. Most treated (1) ________________________________________________
- 2 (2) ________________________________________________
- 3 (3) ________________________________________________
- 4 (4) ________________________________________________
- 5. Least treated (5) ________________________________________________

| Page Break |  |
| --- | --- |

Q39 Prudent (responsible) use of antibiotics in farms involves decreasing unnecessary or inappropriate use of antibiotics. How often do you discuss about prudent antibiotic use with your veterinarian?

- Never (1)
- Sometimes (2)
- About half the time the veterinarian visits (3)
- Most of the time (4)
- Always (5)

Q40 Are the cattle in your farm sometimes treated with antibiotics at dosages higher than the label instructed?

- No (1)
- Not sure (2)
- Yes (3)

Q41 In your farm, who administers the antibiotic medications to the animals? (Check all that apply)

- Producer (1)
- Herdsman (2)
- Milk hand (3)
- Veterinarian (4)
- Manager (5)
- Other (specify) (6) ________________________________________________

| Page Break |  |
| --- | --- |

Q42 What are the appropriate methods for communicating information about prudent use of antibiotics to you? (check all that apply)

- Producer's handbook on prudent use (1)
- Laminated posters (2)
- Videos (3)
- Brochures (4)
- Flow charts for the barn (5)
- Educational seminars (6)
- Other (specify) (7) ________________________________________________

Q43 In what language(s) would you like to be receiving information about prudent use of antibiotics?

- English (1)
- English and Spanish (2)
- Spanish (3)
- Other (specify) (4) ________________________________________________

Q44 Do you use antibiotics for treatment of diseases other than those listed on the bottle/package insert that comes with the medicine)?

- Yes (1)
- Not sure (2)
- No (3)

Display This Question:

If Do you use antibiotics for treatment of diseases other than those listed on the bottle/package in... = Yes

Q45 How is the use antibiotics for treatment of diseases other than those listed on the bottle/package insert done at your farm?

- Based on past use on the farm for treatment of diseases other than those listed on the bottle/package insert (1)
- Based on recommendation of other farmers/producers (2)
- Based on the prescription (written guidelines) from a veterinarian (3)
- Based on my experience as a producer (4)
- Other (specify) (5) ________________________________________________

| Page Break |  |
| --- | --- |

Q46 Do you strictly follow the prescribed course of treatment for each antibiotic medication?

- Never (1)
- Sometimes (2)
- About half the time (3)
- Most of the time (4)
- Always (5)

Q47 Does your farm have written protocols (plans) for treating sick animals with antibiotics?

- Yes (1)
- Not sure (2)
- No (3)

Q48 Is a veterinarian's advice sought before administering antibiotics?

- Never (1)
- Sometimes (2)
- About half the time (3)
- Most of the time (4)
- Always (5)

| Page Break |  |
| --- | --- |

Q49 Which of one the following best describes your opinion about the label instructions for antibiotic medicines?

- Label instructions are difficult to understand and interpret (1)
- Label instructions are easy to understand and interpret (2)

Q50 In what language(s) would you prefer label instructions for antibiotic medicines to be written?

- English (1)
- Spanish (2)
- English and Spanish (3)
- Other (specify) (4) ________________________________________________

Q51 To what extent does the consumer demand for antibiotic-free products influence your use of antibiotics?

- Not at all (1)
- A little (2)
- To a moderate extent (3)
- Quite a bit (4)
- Very much (5)

| Page Break |  |
| --- | --- |

Q52 Which of the following best describes your number of years in cattle farming?

- < 5 years (1)
- 6 - 10 years (2)
- 11 -15 years (3)
- 16 - 20 years (4)
- 21 - 25 years (5)
- 26 - 30 years (6)
- > 30 years (7)

| Page Break |  |
| --- | --- |

Q53 Were you raised on a livestock farm?

- No (1)
- Yes (2)

Q54 Which of the following best describes your gender?

- Male (1)
- Female (2)
- prefer not to answer (3)

Q55 Which of the following best describes your education level attained?

- No school (1)
- Elementary (2)
- Junior high (3)
- High school (4)
- General Education Development (GED) (5)
- Vocational (6)
- College (7)
- Professional (8)
- Other (specify) (9) ________________________________________________

| Page Break |  |
| --- | --- |

Q56 Which of the following best describes the number of cattle in your production unit?

- 1 - 49 (1)
- 50 - 99 (2)
- 100 - 149 (3)
- 150 - 199 (4)
- 200 - 299 (5)
- 300 - 399 (6)
- 400 - 499 (7)
- 500+ (8)

Q57 Which of the following best describes your age group?

- 19 years and below (1)
- 20 - 29 years (2)
- 30 - 39 years (3)
- 40 - 49 years (4)
- 50 - 59 years (5)
- 60 -69 years (6)
- 70 -79 years (7)
- 80+ years (8)

Q58 Any additional comments/recommendations?

________________________________________________________________

________________________________________________________________

________________________________________________________________

________________________________________________________________

________________________________________________________________

End of Block: Default Question Block
